# *Salmonella enterica* Serovar Minnesota Biofilms, Susceptibility to Biocides, and Molecular Characterization

**DOI:** 10.3390/pathogens10050581

**Published:** 2021-05-11

**Authors:** Roberta Torres de Melo, Taciano dos Reis Cardoso, Phelipe Augusto Borba Martins Peres, Raquelline Figueiredo Braz, Guilherme Paz Monteiro, Daise Aparecida Rossi

**Affiliations:** Faculdade de MedicinaVeterinária, Universidade Federal de Uberlândia, Uberlândia 38402-018, Brazil; tacianoreis@hotmail.com (T.d.R.C.); lipe-peres1@hotmail.com (P.A.B.M.P.); llinevet@gmail.com (R.F.B.); guil.paz@hotmail.com (G.P.M.); daise.rossi@ufu.br (D.A.R.)

**Keywords:** biofilms, PCR, PFGE, resistance, *Salmonella*

## Abstract

The presence of virulence genes, phylogenetic relationships, biofilm formation index (BFI), and ultrastructure in *S.* Minnesota at different temperatures (4, 25, and 36 °C) were analyzed. In addition, the ability of biocidal agents (chlorhexidine1%, sodium hypochlorite 1%, and peracetic acid 0.8%) to inhibit biofilms formed by 20 strains isolated from broiler slaughter plants from two Brazilian companies in 2009, 2010, and 2014 was determined. The presence of specific genes was evaluated by PCR and phylogeny between strains by pulsed-field gel electrophoresis. The BFI was determined using tryptone soy broth with 5% of chicken juice, and its structure was observed by scanning electron microscopy. The presence of specific genes indicated that *S*. Minnesota has the potential to cause disease in humans, adapting to adverse conditions. Temperatures of 25 and 36 °C favored biofilm formation, although at 4 °C, there was still biomass that could contaminate the final product. Tolerance to all biocides was identified in 12/20 (60%), representing a real risk of adaptation mechanisms development, especially regarding to resistance to sodium hypochlorite. Phylogenetic analysis indicated cross-contamination and spread among companies, which was probably related to biofilms formation. Results show the necessity of attention to this serovar considering its resistance to sodium hypochlorite, including the need for rigorous control, adopting low temperatures to prevent biofilms formation in the poultry industry.

## 1. Introduction

The genus *Salmonella* comprises a group of zoonotic microorganisms frequently associated with foodborne diseases, having as the main source of human infection the ingestion of contaminated chicken meat [1,2]. The presence of the serotype *S.* Minnesota is already a reason for sanitary embargoes or international warnings due to compromised meat quality related to food security [3,4,5].

Brazil is one of the largest producers and exporters of chicken meat in the world [6]. Information about resistance to biocide agents, virulence factors of strains, its genetic diversity, and their ability to form biofilms in an industrial environment are extremely important, considering the difficulty of *Salmonella* control and the constant need for knowledge about the emergence of this microorganism in the country, which can contribute to the strategic development of measures for the control and prevention of salmonellosis at a global level [7,8,9,10].

In particular, serovars. Minnesota has been prominent among serovars due to the increasing number of isolations in the poultry production chain, prompting research to discover its danger as an agent of human salmonellosis. In Brazil, this serovar was of the second highest incidence between 2007 and 2011 in an investigation of 12,582 strains obtained from chicken carcasses and poultry products [3,11,12].

The participation of *S.* Minnesota in human infection and the strategies that this agent uses for its maintenance in the poultry chain is still little known, so more studies focused on the epidemiological characterization through phylogenetic analysis to understand its spread are necessary. Pulsed-field gel electrophoresis (PFGE) subtyping provides information that favors genotypic characterization, molecular grouping, and subsequently the creation of measures for its control, and it is particularly useful for defining clonal strains and their spread in different areas of industry or for different companies. This information provides the necessary support to trace the source of the problem and determine the most effective actions [13].

Considering the complexity of *Salmonella* and the host/environment interactions, investigations of virulence factors and their persistence in the environment via genotypic and phenotypic analysis allow a better understanding of the pathogen. The identification of genes associated with apoptosis (*avr*A), oxidative stress (*sod*C), invasion (*inv*A), adhesion and biofilm (*agf*A, *sef*A and *lpf*A), and quorum sensing (*lux*S) [14,15] helps to characterize the pathogenic potential and to understand the strategies for perpetuation in the environment. At the same time, its ability to form biofilms stands out; this hinders its control in the food industry [16]. In this condition, microorganisms have different responses to heat treatments, biocides, and antimicrobials, and are therefore a constant source of contamination [17,18].

Knowing the emergence of *S.* Minnesota, the aim of this work was to evaluate the presence of virulence and environmental adaptation genes, the ability of biofilm formation under different conditions, and to establish the phylogenetic relationship of 20 strains of *S.* Minnesota and to elucidate the spread and potential danger that they may represent for human health together with possible control measures of the sessile structure.

## 2. Results and Discussion

### 2.1. Virulence Gene Characterization

The 20 *S.* Minnesota strains showed different frequencies of positivity for the studied genes. Genes linked to apoptosis induction in infected cells (*avr*A), oxidative stress (*sod*C), and invasion (*inv*A) were present in 100% (20/20) of them. Those linked to adhesion (*agf*A), potential biofilm-forming capacity (*lpf*A), and quorum sensing (*lux*S) were identified in 95% (19/20), 75% (15/20), and 80% (16/20), respectively. The strains were discriminated into four distinct virulence profiles (P1, P2, P3, and P4). P1: positive for all genes (10/20–50.0%); P2: negative for *lux*S (4/20–20.0%); P3: negative for *lpf*A (5/20–25.0%); and P4: negative for *agf*A (1/20–5.0%), Table 1.

The detection of *avr*A, *sod*C, and *inv*A genes was unanimous. For *inv*A, this agrees with [19], who analyzed 237 strains of *Salmonella* spp. from food in Brazil and observed *inv*A gene in all of them. According to Wang et al. [20], the *inv*A seems to be very conserved in *Salmonella* spp., justifying its high occurrence.

All strains demonstrated the potential to survive under oxidative stress (*sod*C) and apoptosis induction in infected cells (*avr*A). The existence of these virulence mechanisms reinforces the pathogenic potential of the strains and, despite the absence of reported cases of salmonellosis by this serovar, demonstrates the possibility of its causing disease in humans.

Borges et al. [15] evaluated in 2013, 84 strains of *S*. Enteritidis isolated from 1996 to 2010 in Brazil and observed similar results, with the presence of the *inv*A and *avr*A genes in 100% of the isolates and the *Ipf*A and *agf*A genes in 99% (83/84) and 96% (81/84), respectively. In 2016, Ahmed et al. [21] also observed similar results in Egypt in 20 isolates of *S.* Typhimurium from chicken and in 10 isolates of human origin, with frequency of 100% of *inv*A and *avr*A genes (30/30). They concluded that a high frequency of these genes is observed in serovars, and by that, it could represent a potential cause of salmonellosis in humans.

The absence of the *sef*A gene may be justified according to Amini et al. [22], who observed this specific being restricted to group D *Salmonella,* by classification according to the epizootiology and pathogenicity of different serotypes of *Salmonellaenterica* subspecies. Although not present in all strains, the genetic potential to form biofilms associated with the presence of *agf*A, *lpf*A, and *lux*S genes was observed in 75% (15/20) of the strains. Similar studies with different *Salmonella* serovars have shown the presence of the *agf*A gene in over 91.4%, the *lfp*A gene in 80.63%, and the three genes together in more than 73.34% of strains isolated from birds. The frequency of virulence determinants and the establishment of genetic profiles of isolates help to determine effective control protocols and prevention measures in the industry [15,23,24].

### 2.2. Biofilm Formation × Temperature

All strains (100%) were unable to form biofilm at 4 °C, according to classification by Naves et al. [25] (Table 1). Temperature increase (25 and 36 °C) favored sessile life forms, with the formation of weak biofilms (6/20 strains at 25 °C and 8/20 strains at 36 °C) and medium intensity (3/20 strains at 25 °C and 36 °C; Table 1). No strong BFI was identified at any of the tested temperatures.

These findings agree with of Dhakal et al. [26], who found optical density values equal to those of the negative control for six *Salmonella* serovars tested when kept at refrigeration temperature. However, a study conducted with a standard *S*. Minnesota strain showed that this serovar has the capacity to form medium-intensity biofilms under in vitro conditions using a traditional methodology [18,25].

The grouping of strains using the different BFI classification criteria identified at different temperatures (4, 25, and 36 °C) shows the diversity of biofilm formation dynamics among strains tested under the evaluated thermal conditions and allowed us to classify them into BFI profiles. Six BFI profiles were identified (A–F), the most prevalent being those classified as nonexistent at the three temperatures (9/20–45.0%; Table 1). The BFI classification for this serovar is strain-dependent, and there are probably other factors determining this variability, such as the presence and expression of genes linked to biofilm formation (*agf*A, *lpf*A, and *lux*S) that encode aggregative fimbriae and quorum sensing systems, whose functions favor the biofilm formation [24]. All strains had at least one of these genes, indicating the potential for sessile life that was expressed differently and according to external factors and the expression of this potential.

**Figure 1:**
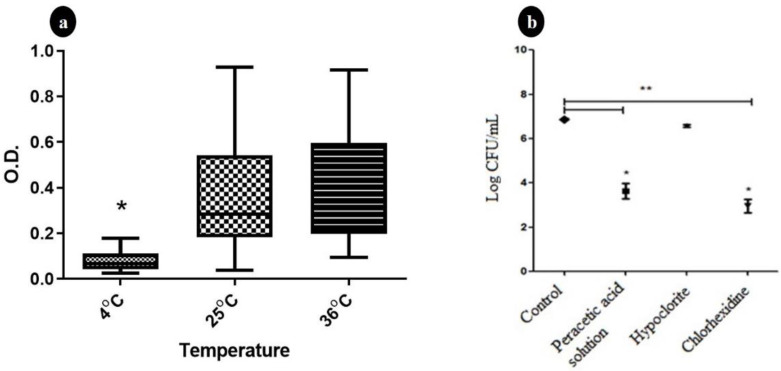
Biomass behavior of 20 *S.* Minnesota strains at different temperatures (**a**) and the counts (Log CFU.mL^−1^) before and after maintenance for 15 min in 0.8% peracetic acid solution, 1% sodium hypochlorite, and 1% chlorhexidine (**b**). O.D.: Optical density at 600 nm. Error bars indicate the standard deviation for the mean O.D. values obtained for each strain; * *p* < 0.001 in comparative analysis between treatments using one way.Anova (**a**). * *p* < 0.001 for counts on samples from the same treatment; ** *p* < 0.001 in the comparative analysis between control and treatments using Kruskal–Wallis (**b**).

Temperatures of 25 °C and 36 °C favored the formation of *S*. Minnesota biofilms at the same intensity compared to a temperature of 4 °C (Figure 1a). The increase in biofilm intensity was related directly to the increase in temperature. This is consistent with Borges et al. [15], who studied biofilms of *Salmonella* spp. in 2013, at temperatures of 3, 12, 28, and 37 °C, whose BFIs ranged from nonexistent to moderate. Although thermal stress at low temperatures is an important trigger for the production of biofilms [27], the test at 4 °C may have made bacterial multiplication unfeasible due to its proximity to the minimum growth temperature [28].

Even under thermal stress conditions at low temperatures, *S.* Minnesota is still capable of forming a biomass allowing its viability, and representing a potential contamination. Considering the high prevalence of the microorganism in Brazilian broiler and poultry slaughterhouses [5], it is possible to suggest its permanence in the industrial environment, even under stress conditions, through the production of biofilm. This should warn the poultry industry to adopt more rigorous control measures for this agent.

Regarding the biofilm formation intensity, the same behavior was identified at temperatures of 25 and 36 °C; these findings contradicts the literature, which states that temperatures below the optimal growth and close to that of the environment intensify the biomass production in *Salmonella* biofilms [29]. According to Čabarkapa et al. [30], factors involved in biofilm production have different responses depending on bacterial strain and according to the temperature of incubation.

Significant differences (*p* < 0.05) considering the BFI within the same serotype indicate that there is probably an influence of intrinsic characteristics, such as the presence of fimbriae, flagella, membrane proteins, and others [31], which even at the molecular level may vary in their expression. Thus, environmental conditions alone are not decisive in the formation of biofilms. Genotypic diversity and the way those factors are expressed also influence the production of sessile biomass by *S.* Minnesota.

**Figure 2:**
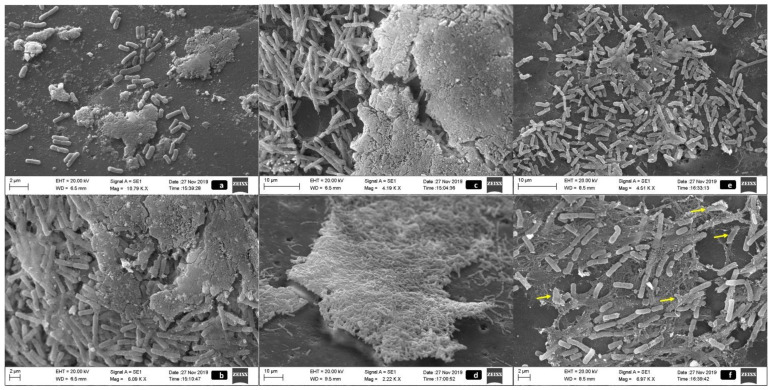
SEM images of biofilms production of three *S.* Minnesota strains at temperatures of 4 °C (**a**) and 36 °C (**b**), and after treatment with different sanitizing agents. (**c**): negative control treated with water. (**d**): treatment with 1% sodium hypochlorite, with maintenance of the structure of the mature biofilm. (**e**): treatment with 1% chlorhexidine. (**f**) (yellow arrows): morphological alteration and loss of membrane integrity in biofilm of *S.* Minnesota treated with 0.8% peracetic acid.

Figure 2a,b allow confirmation of the differences identified in the biofilm according to the temperature. The ultrastructure of the biofilm formed by *S.* Minnesota at temperatures of 25 and 36 °C showed a more stable and mature conformational characteristic compared to that at 4 °C, which is consistent with that found in different *Salmonella* serovars [18].

### 2.3. Performance of Chemical Agents in Sessile S. Minnesota

All chemical agents tested reduced sessile *S.* Minnesota counts after exposure for 15 min. However, significant differences were observed only for peracetic acid and chlorhexidine. In additiontoreducing the counts in 8/20 (40.0%) strains, no growth was observed after the test. Figure 1b illustrates the quantitative results obtained from untreated biofilms, which shows a mean value of 6.87 ± 0.38 log CFU/mL (*p* > 0.05) and from biofilms treated with different products, which showed growth after 15 min contact.

All strains (20/20–100.0%) showed resistance to 1% sodium hypochlorite, so the mean count (6.57 ± 0.55 log CFU/mL) did not differ from that obtained in the control group (*p* > 0.05). The use of peracetic acid or chlorhexidine gave the same results, reducing the counts significantly for resistant strains. The mean counts after treatments were 3.63 ± 2.84 and 2.96 ± 2.55 log CFU/mL, respectively. Those results indicated an average decrease of 3.24 and 3.91 log cycles, respectively, compared to the control group. For both, reductions in the numbers of CFU varied significantly (*p* < 0.001) between tolerant strains. Four chemical resistance profiles were identified, with profile IV 12/20 (60%) showing resistance to all agents. This profile showed negative association when compared with the profile A, which is linked with strains with nonexistence biofilm classification (*p* < 0.0001—Fischer’s test—infinity *odds ratio*) (Table 1).

Peracetic acid, sodium hypochlorite, and chlorhexidine are chemical agents widely used in the cleaning of slaughterhouses. However, the use of peracetic acid and chlorhexidine demonstrated the same efficiency in significantly reducing counts for resistant strains, which is different from that observed for sodium hypochlorite, which showed resistance in all strains. This is alarming because sodium hypochlorite is one of the most widely used cleaning and disinfecting agents in the industry.

Studies performed in Australia and Brazil on chicken meat processing plants showed the efficiency of several chemical agents, including sodium hypochlorite, in reducing the microbial load of *Salmonella* and *Campylobacter* when used properly [32,33]. The strains identified in our study that were tolerant to different sanitizers suggests the inappropriate use of these agents in the routine of the industrial environment, posing a real risk of resistance and adaptation of these bacteria [34]. This can occur because of intrinsic factors of the bacteria, and the resistance may be due to repeated exposure to the agent or developed through genetic modifications [35]. In addition, there may be the appearance of cross-resistance and co-resistance between strains, from the initial resistance to a disinfectant compound, which is followed by the consequent resistance to another agent [35,36,37,38]. It can also be associated with the involvement of molecular factors, such as *RpoS* and *Dps* genes, which are linked to oxidative stress. These genes are actively expressed in *S.* Enteritidis SE86, which is resistant to the presence of sodium hypochlorite at 200 ppm [39]. It is also possible that for *S.* Minnesota, there are similar mechanisms at work in this process. In addition, the properties of this sanitizer can be altered according to pH and the presence of organic matter that alters the availability of hypochlorous acid, reducing its efficiency [40].

Variations in counts for different strains identified after contact with peracetic acid and chlorhexidine demonstrated that the persistence of these microorganisms may be strain-dependent. The existence of strains with a profile of resistance to all agents (profile IV; 12/20–60%; Table 1) indicates that there are probably intrinsic or extrinsic adaptive mechanisms allowing their survival. Although the use of chemical compounds brings benefits in disinfection, these agents usually have limitations, such as not destroying residual structures of the bacterial biofilm matrix, facilitating the resurgence or even maintaining these structures on surfaces [41]. We observed that the external matrix was maintained only when strains were treated with sodium hypochlorite. Thus, special efforts are required for the complete removal of *S*. Minnesota biofilms adapted to this biocide. It is probable that effectiveness in controlling these microorganisms will be achieved through sanitation plans combining cleaning measures focused on the elimination of the extrapolymeric matrix and using different agents in a periodic rotation.

In a different way, peracetic acid and chlorhexidine proved to be effective in eliminating the external matrix and disrupting the conformation of the mature biofilm. This profile may be associated with a mechanism of action aimed at the denaturation of proteins, cellular enzymes, increased permeability of the bacterial cell, and cell lysis [42].

### 2.4. Ultrastructure of Treated Biofilms

By SEM, mature biofilm was not characterized when the bacteria were kept at a temperature of 4 °C (Figure 2a). As in the microbiological test, the biofilm formed at this temperature was of low intensity, either at the start of its development process or as a result of a possible stagnation in the initial stages, and of which we detected only the presence of punctual microcolonies in the field. Temperatures of 25 and 36 °C (Figure 2b) allowed the development of dense clusters of bacteria with evident matrix production between the bacteria and in the outermost region providing the necessary protection to the bacterial community.

The biomass was maintained for the three strains and showed similar characteristics for all (Figure 2c–f). Figure 2c illustrates the maintenance of the integral structure of the biofilm without alteration in its three-dimensional conformation, which is highlighted by the bacterial agglomeration. This pattern was identified in water-treated biofilms for the three strains. The negative control allowed the characterization of the biofilm formed by this serovar and showed a denser and more stable architecture and presented a compact coverage along the surface associated with the presence of exchange channels.

The external cover of extracellular matrix was a less evident parameter in bacteria after treatment with sodium hypochlorite, but the maintenance of macro colonies showed a matrix connecting bacteria with the contact surface, which is a characteristic of a mature biofilm (Figure 2d). The treatment with chlorhexidine (Figure 2e) and peracetic acid (Figure 2f) promoted not only the reduction of the external EPS matrix but also a weakening of the connection between the bacteria and a greater access of the microorganism to the chemical agent, which is consistent with the logarithmic reduction that was identified in the counting analyses. The existence of dispersed groups of bacterial aggregates evidenced a loss of biofilm conformation and cellular viability or still immature biofilms with primary production of an extracellular matrix. Some of the bacterial cells showed altered morphology and impaired membrane integrity (Figure 2f).

### 2.5. Phylogenetic Analysis

**Figure 3:**
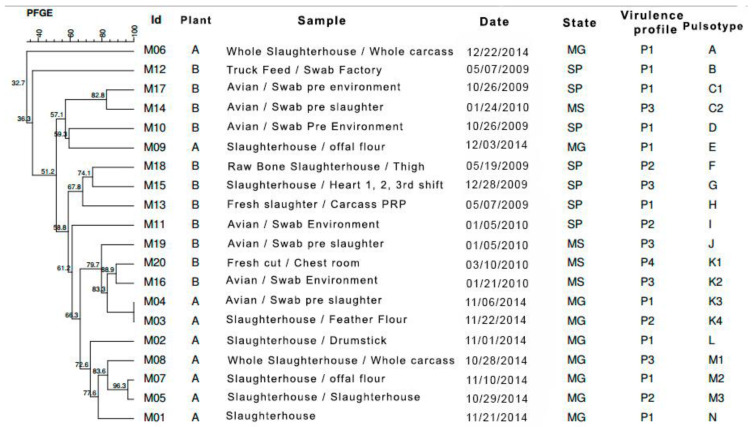
Comparative dendrogram of 20 *S.* Minnesota strains, constructed from PFGE results considering the isolation location, date of collection, and the presence or absence of *avr*A, *sod*C, *inv*A, *agf*A, *lpf*A, and *lux*S genes using the Dice similarity coefficient with 1.5% tolerance and UPGMA method with 0.80% optimization. Presence of 14 pulsotypes (A to N)—three showed genotypic similarities above 80% “C”, “K”, and “M”.

The dendrogram constructed from the PFGE results was compared considering the isolation location, date of collection, and the evaluated genotypic and phenotypic characteristics. Similarity analysis of the 20 strains of *S.* Minnesota presented pulsotypes (A–N; Figure 3), in which three groups, C, K, and M, showed genotypic similarities above 80%.

Only pulsotypes C, K, and M contained strains that allowed an epidemiological evaluation. Pulsotype C contained two strains, M17 and M14, with a similarity of 82.8%, coming from the industry B in 2009 and 2010 and having the *avr*A, *sod*C, *inv*A, *agf*A, and *lux*S genes in common. Although they were from the same industry (B), they were from distinct flocks, São Paulo and Mato Grosso do Sul, indicating possible dissemination of this profile by transport.

Pulsotype K contained four strains, two of them (M04 and M03) being clones isolated from the slaughterhouse of industry A in Minas Gerais, Brazil. Cross-contamination may explain the occurrence of this profile, since they were isolated at closely separated times, both dated November 2014. The presence of *avr*A, *sod*C, *inv*A, *agf*A, and *lpf*A genes was common to both.

Other strains of the K (M20 and M16) pulsotypes were isolated in 2010: both in the aviary and in the industry B cutting room in Mato Grosso do Sul, Brazil. The *avr*A, *sod*C, *inv*A, and *lux*S genes and resistance to sodium hypochlorite and peracetic acid in biofilms were common. These data indicate that the maintenance of these microorganisms in sessile form and contamination of the final product may be caused by cross-contamination [11].

There were also signs of neglect of biosecurity standards in the food industry environment due to the presence of the agent in clean and dirty processing areas. According to Moura et al. [43], this oversight is decisive in the maintenance of the microorganism in the environment. At the same time, pulsotype K indicated that there was spread of this profile, since there were similar strains in Mato Grosso do Sul and Minas Gerais, Brazil, in different slaughter units.

The pulsotype M contained three strains (M08, M07, and M05) with 83.6% of similarity to 2014, coming from the slaughterhouse of industry A in Minas Gerais, Brazil. The strains presented the *avr*A, *sod*C, *inv*A, and *agf*A genes in common.It is possible to suggest that strains with homology greater than 80% persist in both the poultry and slaughterhouse environment, which is probably due to the presence of biofilms and, even if identified at low intensities in our assays, demonstrated the viability of the sessile bacterial strains at high counts, even in the presence of biocidal agents.

The presence of virulence characteristics associated with the high phylogenetic diversity of the strains indicates the zoonotic potential of this serovar and the possible risks associated with its ability to adapt due to its genetic plasticity. The ability to produce biofilms combined with resistance to chemical agents reveals the need for special attention to this serovar in chicken production, with a view to preventing, controlling, and constantly monitoring the sessile structure and its tolerance to sanitizers.

## 3. Materials and Methods

### 3.1. Samples and Sampling

Twenty *S.* Minnesota strains isolated from 2009, 2010, and 2014 were used, originating from broiler slaughtering plants of two Brazilian companies (A and B) with complete production cycles and integration systems inspected by the Federal Inspection Service SIF and qualified for internal and external trade. From company A, located in the state of Minas Gerais, nine isolates were used and from industry B, with slaughtering plants in the states of São Paulo and Mato Grosso do Sul, seven and four isolates were used, respectively.

Isolations were performed in industrial laboratories, with samples collected at previously established points in internal control programs and/or required by Bacteriological Analytical Manual, from U. S. Food and Drug Administration (BAM/FDA) (FDA, 2007). The strains were assigned to the study after biochemical identification, as recommended by the same official protocol [44] and serological analyses (Oswaldo Cruz Institute Foundation in the State of Rio de Janeiro—IOC/FIOCRUZ, Rio de Janeiro, Brazil), and were stored in the collection of the Applied Animal Biotechnology Laboratory of the Federal University of Uberlândia (LABIO/UFU, Minas Gerais, Brazil).

### 3.2. Reactivation of Strains and Extraction of Genomic DNA

The strains were obtained from pure cultures maintained on nutrient agar (AN–OXOID^®^, Roskilde, Denmark) under refrigeration and reactivated in brain heart infusion (BHI–OXOID^®^) broth followed by subculturing on tryptone soy agar (TSA–OXOID^®^), respecting the conditions of incubation at 37 °C for 24 h.

Bacterial suspension obtained in tryptone soy broth (TSB–OXOID^®^) overnight was used for the extraction of genomic DNA using the Wizard^®^ Genomic DNA Purification commercial kit (Promega^®^, Madison, WI, USA).

### 3.3. Identification of Specific Genes

Conventional PCR was used to identify genes linked to the apoptosis process (*avr*A), potential survival under oxidative stress (*sod*C), invasion (*inv*A), adhesion, and biofilm formation (*agf*A, *sef*A, and *lpf*A) and quorum sensing (*lux*S) (Table 2).The PCR reaction preparation consisted of 12.5 µL of GoTaq^®^ Green Master Mix (Promega^®^), 1 µL of DNA at 10 ng/µL, 1 µL of the gene-specific *primer* pairs (Table 2), and 10.5 µL of Milli-Q^®^ Water (Merck^®^, Darmstadt, Germany). The microtubes were transferred to a thermal cycler (Eppendorf^®^, Hamburg, Germany) for amplification: an initial denaturation cycle at 94 °C for 5min, 35cycles of denaturation at 94 °C for 45 s, annealing for 30 s at 58 °C (*inv*A); 50 °C (*sef*A and *1pf*A), 66 °C (*agf*A) or 62 °C (*avr*A, *sod*C and *lux*S); extension at 72 °C for 90 s, and a final extension at 72 °C for 10 min. The positive reaction control used was the strain *S.* Enteritidis ATCC13076 and, as negative control, sterile ultrapure water. Agarose gels (Afllymetrix^®^, Santa Clara, CA, USA) were stained with SYBR^®^ Safe DNA gel stain solution (Invitrogen^®^) and visualized under UV light on a transilluminator (Loccus Biotechnology^®^, São Paulo, Brazil) after 90 min of running the gel at 100 W, 80 V, and 80 A.

### 3.4. Pulsed-Field Gel Electrophoresis (PFGE)

The PulseNet protocol, according to Ribot et al. [49] was used to conduce pulsed-field gel electrophoresis (PFGE). Bacteria grown at 37 °C overnight on TSA (OXOID^®^) were suspended in tubes containing 2mL of phosphate-buffered saline (PBS: 0.01 M phosphate buffer; pH 7.2; 0.85% NaCl). After agarose blocking, genomic DNA digestion was performed with 30 U of XbaI enzyme (Invitrogen^®^) for 2 h at 25 °C.The DNA fragments were separated on 1% agarose gel (SeaKem Gold^®^) in 0.5X TBE buffer in CHEF DRIII (Bio-Rad^®^, Hercules, CA, USA) for 18h with the following parameters: 200 V, 120° angle, 6 V/cm gradient, and 14 °C buffer temperature. The comparison of the band patterns was performed by the UPGMA analysis method, using the Dice similarity coefficient with a tolerance of 1.5% in the comparison of the position of the bands.

### 3.5. Biofilm Formation Index

The determination of biofilm formation index (BFI) was performed according to Kudirkienė et al. [50] and Naves et al. [25], with modifications. The strains, previously selected and serotyped by the supplier company, were cultivated in TSB (OXOID^®^) supplemented with 5% of chicken juice to simulate nutritional stress in a slaughterhouse environment [51]. Incubation was performed for 24 h at 37 °C under constant agitation (6.16 g) until reaching OD_600_close to 0.25. The suspension was centrifuged at 5031× *g* for 10 min at 4 °C, and the pellet obtained was washed with 0.9% NaCl solution in three successive centrifugations. The final wash suspension was added to TSB (OXOID^®^) supplemented with 5% of chicken juice at a 1:100 concentration, which was then added to a polystyrene microplate (Kasvi^®^, Paraná, Brazil) and incubated for 24 h at the three pre-set temperatures: 4, 25, and 36 °C. This procedure was performed individually for each strain. The strain *S.* Enteritidis ATCC13076 was used as control. Three repetitions were performed with eight replicates for each temperature. The biofilm formed at the bottom of the wells was washed twice with 0.9% NaCl solution and dried at 55 °C for 50 min. After drying, 200 µL of 1% crystal violet solution (Synth^®^, São Paulo, Brazil) was added to each well for 5 min. The plates were washed three times with ultrapure water and dried at 55 °C for 15 min, the dye was eluted with methyl alcohol solution (Synth^®^), and the absorbance was read at OD_600_. Results from suspended cells and adhered cells were tabulated to determine the BFI using the formula:

BFI = AB − PC/SB

where BFI is the result of the detected index, AB the reading of adherent bacteria, PC is the absorbance reading of the control wells without microorganisms, and SB is the absorbance reading of the suspended bacteria. They were classified as strong if ≥1.10, medium if 0.70–1.10, weak if 0.36–0.69, or nonexistent if ≤0.35.

### 3.6. Biofilm Formation Inhibition Test

A biofilm formation inhibition test was performed according to Lu et al. [52] individually for each strain. The biofilm formation was performed from a 100 µL of inoculum containing 10^7^ CFU/mL of the bacteria on four cellulose membranes placed in TSA (OXOID^®^) agar plates incubated at 36 °C for 24 h. After incubation, membranes were removed and transferred to a new TSA plate (OXOID^®^), which was incubated under the same conditions and repeated after 24 h. On the third day, membranes were added to sterile vial treatments, the first being the TSB control, the second with 1% chlorhexidine (Vicpharma^®^, São Paulo, Brazil), the third with sodium hypochlorite 1% (Sanikoll^®^, São Paulo, Brazil), and the fourth with 0.8% peracetic acid (Synth^®^). Each treatment was incubated for 15 min at room temperature, respecting the company’s hygiene protocols. Membranes were washed with Letheen broth, and then, 1% trypsin solution (ThermoFisher^®^, Waltham, MA, USA) was added and maintained for 15 min. Serial dilutions of detached content were made in saline solution 0.9% to dilution 10–6, which were added to TSA plates and incubated at 36 °C for 24 h to perform counts.

### 3.7. Scanning Electron Microscopy

For confirmation of phenotypes obtained in biofilm formation assays, three randomly selected strains that showed resistance to the three chemical agents tested were evaluated for the ultrastructure formed at temperatures of 4, 25, and 36 °C and, separately, in treatments with 1% sodium hypochlorite,1% chlorhexidine, or 0.8% peracetic acid after production of the sessile structure at 36 °C. The assays were performed by scanning electron microscopy (SEM).The preparation of material for SEM analysis was according to Brown et al. [53]. Samples were dried on critical point drying (CPD; CPD 030, Baltec^®^, Liechtenstein, Deutsch) using liquid carbon dioxide as transition fluid; then, they were coated with a 20-nm gold layer (SCD 050, Baltec^®^) and examined by SEM (Zeiss Supra 55 FEG) operating at 20 kV.

### 3.8. Analysis of Results

Results of gene virulence were analyzed using descriptive statistics, with calculation of percentages. Analysis for dendrogram construction was performed using GelCompar II software. Interpretation of data related to biofilms was done using GraphPadPrism^®^ software, version 7.0. Simple analysis of variance test (ANOVA) was applied for qualitative and quantitative biofilm formation tests and comparison between treatments. For biofilm inhibition test, a nonparametric Kruskal–Wallis test was used. For all tests, a 95% of confidence interval was used.

## Figures and Tables

**Table 1:** Classification of 20 *S.* Minnesota strains according to biofilm formation index (BFI) at different temperatures and the profiles of biofilm formation (a), virulence (b), and chemical resistance (c).

Strains	4 °C		25 °C		36 °C		Profile (a)	Profile (b)	Profile (c)
	BFI	Class	BFI	Class	BFI	Class.			
M02	0.144	NE	0.226	NE	0.217	NE	A	P1	I
M13	0.025	NE	0.284	NE	0.095	NE	A	P1	I
M17	0.056	NE	0.219	NE	0.206	NE	A	P1	I
M03	0.056	NE	0.175	NE	0.269	NE	A	P2	II
M11	0.037	NE	0.039	NE	0.136	NE	A	P2	II
M18	0.077	NE	0.173	NE	0.196	NE	A	P2	II
M14	0.059	NE	0.179	NE	0.105	NE	A	P3	III
M16	0.039	NE	0.179	NE	0.215	NE	A	P3	III
M20	0.030	NE	0.196	NE	0.118	NE	A	P4	IV
M10	0.039	NE	0.229	NE	0.537	W	B	P1	IV
M01	0.159	NE	0.284	NE	0.916	M	C	P1	IV
M06	0.162	NE	0.471	W	0.431	W	D	P1	IV
M12	0.067	NE	0.517	W	0.421	W	D	P1	IV
M05	0.079	NE	0.493	W	0.438	W	D	P2	IV
M15	0.088	NE	0.576	W	0.644	W	D	P3	IV
M07	0.118	NE	0.550	W	0.866	M	E	P1	IV
M19	0.054	NE	0.462	W	0.875	M	E	P3	IV
M04	0.066	NE	0.891	M	0.539	W	F	P1	IV
M09	0.179	NE	0.928	M	0.547	W	F	P1	IV
M08	0.074	NE	0.870	M	0.612	W	F	P3	IV
Total N (%)	NE: 20 (100)		NE: 11 (55)		NE: 9 (45)		A: 9 (45)	P1: 10 (50)	I: 3 (15)
	W: 0		W: 6 (30)		W: 8 (40)		B: 1 (5)	P2: 4 (20)	II: 3 (15)
	M: 0		M: 3 (15)		M: 3 (15)		C: 1 (5)	P3: 5 (25)	III: 2 (10)
							D: 4 (20)	P4: 1 (5)	IV: 12 (60)
							E: 2 (10)		
							F: 3 (15)		

N (%): Number of strains and their percentage. Class: Classification; NE: nonexistent; W: weak; M: medium. A (NE, NE, NE); B (NE, NE, W); C (NE, NE, M); D (NE, W, W); E (NE, W, M); F (NE, M, W). P1 (*avr*A, *sod*C, *inv*A, *agf*A, *lpf*A, *lux*S); P2 (*avr*A, *sod*C, *inv*A, *agf*A, *lpf*A); P3 (*avr*A, *sod*C, *inv*A, *agf*A, *lux*S); P4 (*avr*A, *sod*C, *inv*A, *lpf*A, *lux*S). I (Hypochlorite); II (Hypochlorite, Acid Peracetic); III (Hypochlorite, Chlorhexidine), IV (Hypochlorite, Acid Peracetic, Chlorhexidine). Gray marking indicates positive correlation (*p* < 0.0001—Fischer test—CI 95% 8.244 to infinity).

**Table 2:** *Primers* used to identify specific genes in *S.* Minnesota strains.

Gene	Concentration	Amplicon (bp)	*Primer*	Reference
*avr*A	20 pmol	385	GTTATGGACGGAACGACATCGG ATTCTGCTTCCCGCCGCC	[45]
*sod*C	20 pmol	500	ATGAAGCGATTAAGTTTAGCGATGG TTTAATGACTCCGCAGGCGTAACGC	[14]
*inv*A	10 pmol	284	GTGAAATTATCGCCACGTTCGGGCAA TCATCGCACCGTCAAAGGAACC	[46]
*sef*A	10 pmol	488	GATACTGCTGAACGTAGAAGG GCGTAAATCAGGATCTGCAGTAGC	[46]
*agf*A	10 pmol	350	TCCACAATGGGGCGGCGGCG CCTGACGCACCATTACGCTG	[47]
*lpf*A	10 pmol	250	CTTTCGCTGCTGAATCTGGT CAGTGTTAACAGAAACCAGT	[47]
*lux*S	20 pmol	1080	GATAATCCTGAACTAAGCTTCTCCGC GGTTATGAGAAAAGCATGCACCGATCA	[48]

bp: base pairs.
